# Progress in Understanding the Physiological and Molecular Responses of *Populus* to Salt Stress

**DOI:** 10.3390/ijms20061312

**Published:** 2019-03-15

**Authors:** Xiaoning Zhang, Lijun Liu, Bowen Chen, Zihai Qin, Yufei Xiao, Ye Zhang, Ruiling Yao, Hailong Liu, Hong Yang

**Affiliations:** 1 Guangxi Key Laboratory of Superior Timber Trees Resource Cultivation, Guangxi Forestry Research Institute, 23 Yongwu Road, Nanning 530002, China; sflzxn@163.com (X.Z.); gfri_bwchen@163.com (B.C.); qinzihai@126.com (Z.Q.); xiaoyufei33@163.com (Y.X.); elaine.ye@163.com (Y.Z.); jullyudi@163.com (R.Y.); 2 Key Laboratory of State Forestry Administration for Silviculture of the lower Yellow River, College of Forestry, Shandong Agricultural University, Taian 271018, Shandong, China; lijunliu@sdau.edu.cn; 3 Key Laboratory of Economic Plants and Biotechnology, Kunming Institute of Botany, Academy of Sciences, Yunnan Key Laboratory for Wild Plant Resources, Kunming 650201, China

**Keywords:** poplars (*Populus*), salt tolerance, molecular mechanisms, SOS, ROS

## Abstract

Salt stress (SS) has become an important factor limiting afforestation programs. Because of their salt tolerance and fully sequenced genomes, poplars (*Populus* spp.) are used as model species to study SS mechanisms in trees. Here, we review recent insights into the physiological and molecular responses of *Populus* to SS, including ion homeostasis and signaling pathways, such as the salt overly sensitive (SOS) and reactive oxygen species (ROS) pathways. We summarize the genes that can be targeted for the genetic improvement of salt tolerance and propose future research areas.

## 1. Introduction

Poplars (*Populus* spp.), which include about 100 species [1], are widely distributed across a variety of climatic regions [2] and have become important species for global afforestation and shelterbelt projects because of their rapid growth and high biomass yields [3]. These traits, in combination with other characteristics, such as extensive and deep root systems, considerable genetic variation, small genome size, convenient asexual propagation, genetic transformability, and economic significance, have led to the use of poplars as model tree species [4,5,6].

The increasing salinization of soils has greatly limited the planting of salt-sensitive *Populus* species [7]. Salt stress (SS) induces water deficiency, osmotic stress, ion toxicity, and oxidative damage [8] and thereby reduces photosynthesis, respiration, transpiration, metabolism, and growth in poplars. Like most plants, poplars can adapt to SS by maintaining their cellular ion homeostasis, accumulating osmotic-adjustment substances, and activating scavengers of reactive oxygen species (ROS) via the initiation of an efficient signal transduction network [9]. Desert poplar (*Populus euphratica*) is one of the most salt-tolerant *Populus* species [10] and is often used to study the salt-response mechanisms of trees. *P. euphratica* was reported to be tolerant to up to 450 mM NaCl (about 2.63%) under hydroponic conditions and showed high recovery efficiency when NaCl was removed from the culture medium [11]. A previous study has shown that *P. euphratica* could grow in soils with up to 2.0% salinity and can survive in soils with up to 5.0% salinity [12]. As a non-halophyte, *P. euphratica* could activate salt secretion mechanisms when soil salinity concentrations are greater than 20%, which may be one of the reasons for its high salt-tolerance [13]. Most other *Populus* species are relatively salt-sensitive, including the grey poplar (*Populus* × *canescens*), *Populus* × *euramericana*, and *Populus popularis*, which are usually used as a salt-sensitive control in salt-response studies. *Populus alba* has a wide variation of salinity tolerance within the species: for example ‘Guadalquivir F-21–38′, ‘Guadalquivir F-21–39′, and ‘Guadalquivir F-21–40′ clones show salt tolerance, while most other clones have a common salt sensitivity. Considering the wide variation within the species, *P. alba* could be used as a model species to understand the mechanisms of SS [2].

Previous research primarily focused on the anatomical, physiological, and biochemical changes in poplars during SS; many recent studies have focused on the molecular mechanisms using new techniques, such as genome-scale transcript analysis [14], high-throughput sequencing [15], metabolite profiling [16], bioinformatic analyses [17,18,19], and a non-invasive micro-test technique (NMT) [20].

Here, we review the recent progress in understanding the physiological and molecular responses of *Populus* to SS, including SS injuries, the main mechanisms of salt tolerance, and the genes targeted for the genetic improvement of salt tolerance in *Populus*, with a major focus on ion homeostasis, osmotic adjustment, ROS scavenging, salt overly sensitive (SOS) signaling pathways, potential candidate genes, and transcription factor-mediated regulation of the SS response. 

## 2. SS Injury 

### 2.1. Inhibition of Poplar Growth by Salinity Stress

Salinity affects all stages of *Populus* growth, including germination [21], vegetation growth [15,22], and sexual reproduction [23]. The percentage of seeds that germinate and the extent of leaf expansion were both reported to decline as salt concentrations increase [21]. In addition, shoot growth is more sensitive to salt than root growth [8]. When salt-sensitive white poplar (*P. alba*) clones were exposed to SS (0.6% NaCl), striking reductions were observed in their leaf elongation rate and internode lengths, while significant increases were observed in the numbers of short branches and bud numbers, as well as in the levels of leaf epinasty, necrosis, and abscission [7]. 

Long-term SS also induces early leaf maturity, early flowering, and early tree maturation in *P. alba* clones, which display a greater architectural modification when exposed to high SS (0.6%) than lower SS (0.3%) [22]. When exposed to 0.6% NaCl, the heights, ground diameter, and leaf numbers of Poplar 107 were significantly reduced, while plants exposed to 0.3% NaCl stress had relatively minor phenotypic changes [15]. When *P. euphratica* was exposed to 300 mM (1.76%) NaCl stress, the three growth indexes (plant height, ground diameter, and leaf number) were reduced to 31%, 45.5%, and 20% of the control plants, respectively. The mean leaf area of these stressed trees was reduced by up to 60%, and the leaves began to wither and yellow after 10 days. By contrast, a treatment of 50 mM (about 0.29%) NaCl did not cause a significant reduction in these traits in *P. euphratica* [24].

### 2.2. Salt-Induced Physiological and Cellular Changes

The adverse effects of SS also result in physiological and microscopic anatomical changes. *P. euphratica* and *P. alba* trees exposed to SS have significantly reduced stomatal area, aperture, and conductance, but increased stomatal density and hydraulic conductance [25,26,27]. The salt-induced reduction of leaf area in *Populus* may be one of the reasons for the increased stomatal density and decreased stomatal area [24,27]. The percentage loss of hydraulic conductivity (PLC%) in *P. euphratica* increased from 31.81% at 0 mM NaCl to 83.83% at 150 mM NaCl (0.88%), causing a 40–80% decrease in hydraulic conductivity and ensuring high hydraulic efficiency [27]. *Populus* trees thus reduce their transpiration by decreasing their stomatal apertures and conductance and increasing their PLC% values to cope with the salt-induced water deficit.

*Populus* may adjust their xylems to adapt to salinity stress [28]; for example, when exposed to SS, salt-sensitive *P. × canescens* produces narrower xylem vessels in which it stores sodium ions (Na^+^), reducing the effect of ion toxicity. In contrast, the salt-tolerant species *P. euphratica* produces narrow xylem vessels to reduce Na^+^ uptake even under normal conditions, the abundance of which remains largely unaltered under moderate SS [29]. Overall, this may indicate an evolutionary adaptation of the xylem structure in *P. euphratica*.

### 2.3. Salt-Induced Damage to the Photosynthetic System

Chlorophyll (Chl) is the main pigment used for photosynthesis in plants and is often studied in the evaluation of salt damage. The contents of Chla and Chlb, as well as the relative electron transport rate, decreased significantly in poplar 107 (a superior variety selected from *Populus × euramericana* cv. ’74/76′ hybrids) under 0.6% NaCl but increased under 0.3% NaCl [15]. 

Almost all poplar trees show a decreased net photosynthetic rate (Pn) under high SS; for example, the Pn decreased by 48.3% in *P. euphratica* under a 300 mM NaCl treatment, in comparison with the unstressed plants [24]. SS has a negative effect on growth, possibly due to the Pn affecting the accumulation of biomass. The stomatal conductance (Gs), transpiration rate, and internal CO_2_ (Ci) concentration of *P. euphratica* leaves also decreased by 48.5%, 42.1%, and 15.7% under 300 mM NaCl, indicating that the photosynthetic system was injured [24]. The fluorescence transient curve OJIP is highly sensitive to salinity stress; the OJ phase is the photochemical phase, leading to the reduction of QA to QA^-^. The I level is related to the heterogeneity in the filling up of the plastoquinone (PQ) pool. The P level is reached when all the PQ molecules are reduced to PQH2 [30]. While in poplars treated with 0.3% NaCl the OJIP curve follows the same trend as in the control plants, it significantly decreases at the J, I, and P phases in plants treated with 0.6% NaCl [15]. Large decreases in the J and I phases are also observed in *P. euphratica* treated with 300 mM NaCl [24]. As the electron transfer between Pheo and QA occurs during the J phase, these results indicate the higher salt concentration had a significant influence on electron transfer, which led to a great degree of salt damage [15]. Besides, F_0_ (minimal constant fluorescence of dark-adapted plants) and Fv/Fm (a surrogate of the maximum quantum efficiency of PSII) are also used as fluorescence parameters to identify salt tolerance, for example, to detect genotypic differences in the sensitivity of white poplar clones to SS [31]. 

## 3. Primary Mechanism of Salt Tolerance in *Populus*

### 3.1. Maintaining an Optimal K^+^/Na^+^ Ratio

Salt tolerance can be determined by the net Na^+^ efflux capacity in *Populus*. The net uptake of Na^+^ depends on its influx, exclusion, and sequestration, as well as on other Na^+^ regulation processes, such as xylem Na^+^ loading and unloading, and phloem Na^+^ recirculation [32]. Na^+^ moves into cells through non-selective cation channels (NSCCs) and high-affinity K^+^ transporter (HKT) [33], while excessive Na^+^ can be extruded into the apoplast across the plasma membrane (PM) by the Na^+^/H^+^ antiporter salt overly sensitive1 (SOS1; see Section 3.4) [34], which may also involve Na^+^ loading in the xylem [35]. The net Na^+^ efflux increased significantly in *P. euphratica* after 0.5–12 h under 100 mM NaCl but was reduced at 0.5 h in *P. popularis*, which showed an overall Na^+^ influx after 6–12 h of SS [36]. This is consistent with results of X-ray microanalysis showing that *P. euphratica* had lower concentration of Na^+^ in all subcellular compartments than salt-sensitive poplar species [37]. Moreover, salt tolerance can be improved by increasing Na^+^ efflux and the uptake of mineral nutrients via interactions between mycorrhizal fungi and the roots of salt-sensitive *Populus* [10,38]. 

It is crucial for *Populus* to maintain an optimal K^+^/Na^+^ ratio in the cytoplasm when exposed to high salinity. An excessive uptake of Na^+^ ions not only increases their abundance in plant cells, but also induces the loss of potassium ions (K^+^) by depolarizing the cellular membranes. Moreover, Na^+^ can compete with K^+^ for the binding sites of the uptake system, resulting in an imbalance of the K^+^/Na^+^ ratio that eventually causes ion toxicity [32]. Under high SS, *P. euphratica* maintained an optimal Na^+^/K^+^ ratio by restricting the net Na^+^ uptake and transport from roots to shoots and by maintaining higher K^+^ uptake and transport [39,40]. X-ray microanalysis showed that high salinity reduced the K^+^/Na^+^ ratio by 93% in *P. popularis* but only by 69% in *P. euphratica* [40]. 

*Populus* improves salt tolerance by accumulating Na^+^/K^+^ ions in the vacuoles. Na^+^ is sequestered into plant cell vacuoles by the tonoplast Na^+^/H^+^ antiporter NHX1 [41]. Besides detoxifying the cytoplasm, the accumulation of Na^+^ ions in the vacuoles is used as an osmoticum to draw water into the cells [42]. Vacuolar H^+^-ATPase and vacuolar H^+^-PPase generate an electrochemical gradient across the vacuolar membrane for the tonoplast Na^+^/H^+^ antiporters in *P. euphratica* cells [42]. In addition, expressing the *Arabidopsis thaliana* Na^+^/H^+^ antiporter gene *AtNHX1* in transgenic poplars improves their salt resistance by improving their Na^+^/H^+^ exchange activity [43,44,45]. AtNHX3 was previously shown to act as the tonoplast K^+^/H^+^ antiporter for the transportation of K^+^ and the maintenance of ion homeostasis [46]; however, AtNHX1 was also recently found to enhance the accumulation of K^+^ in the vacuoles of transgenic poplars [47]. Yang reported that the constitutive expression of either *AtNHX1* or *AtNHX3* in transgenic *Populus* increased the vacuolar accumulation of Na^+^ and K^+^, leading to improved salt tolerance and drought tolerance [47]. 

K^+^ and Na^+^ uptake and transport mediated by HKT1 facilitate the rapid response of *Populus* to SS. The Na^+^/K^+^ transporter HKT1 is located on the plasma membrane and mediates the uptake and transport of K^+^ and Na^+^ in poplar. Furthermore, HKT is also involved in xylem Na^+^ unloading in tomato (*Solanum lycopersicum*) [48], as well as in the phloem-mediated recirculation of Na^+^ from the shoots to the roots of rice (*Oryza sativa*) [49], avoiding the excessive accumulation of Na^+^ in the leaves. The expression of *HKT1* in *P. euphratica* is three times higher after 1 h of a 1% NaCl treatment, which facilitates its rapid response to SS by taking a certain amount of Na^+^ ions into the cells to maintain their osmotic balance [50].

Restricting the K^+^ efflux is important for the salt resistance of *Populus.* Plasma membrane H^+^-ATPase restricts K^+^ efflux, thus improving salt resistance of *Populus*. K^+^ can be transported into cells via an inward-rectifying K^+^ channel and the high-affinity K^+^ transporter HKT1, while K^+^ efflux from the roots is mediated by the activation (by depolarization) of outward-rectifying K^+^ channels (DA-KORCs) and NSCCs (DA-NSCCs), which can be induced by salt and inhibited by the plasma membrane H^+^-ATPase [51,52]. The concentration of K^+^ is markedly reduced in *P. euphratica* callus cells exposed to SS when pretreated with vanadate, an inhibitor of H^+^-ATPase, because of the enhanced efflux of K^+^ [53,54]. 

H^+^-ATPase activity plays a large role in salt tolerance of *Populus*. In addition to restricting K^+^ efflux, H^+^-ATPases can also maintain a proton gradient across the membrane, used by the Na^+^/H^+^ antiporters [8,55,56], which is closely associated with the salt sensitivity of *Populus*. The activity of plasma membrane H^+^-ATPase was higher in salt-tolerant genotypes than in salt-sensitive genotypes of *P. alba* [57]. Similarly, H^+^-ATPase genes were more highly expressed in the salt-tolerant species *P. euphratica* than in the salt-sensitive *Populus trichocarpa* [55]. This is further supported by the work of Ma et al., who showed that the *P. euphratica* genome contains more copies of the P-type H^+^-ATPase genes than *P. trichocarpa* [58].

Overall, except the above mechanisms, *P. euphratica* owns more effective mechanisms to respond to SS, for example, develop smaller vessel lumina than other salt sensitive poplars to limit ion loading into the xylem and develop leaf succulence after a long time of SS to dilute salt as a plastic morphological adaptation. Up to one-fifth of Na^+^ and one-third of Cl^−^ are stored in foliage in the harvest season and are eliminated as leaves are ultimately shed. Excessive Na^+^( Cl^−^) can also be extruded via phloem retranslocating into the roots [59]. Sequestering Cl^−^ in cortical vacuoles at high salinity is also important for restricting its transport into above-ground organs [60].

### 3.2. Accumulation of Osmotic-Adjustment Substances

The water potential of the soil and the availability of water to plant roots are lower in saline soils; therefore, salt-stressed plants first experience water deficiencies caused by osmotic stress [16]. Stress caused by 150 mM NaCl caused a drop of −0.68 MPa in the osmotic potential and a rapid decrease of 0.77 MPa in the shoot water potential in young *P. euphratica* trees [16]. Plant cells tend to accumulate soluble osmolytes to adjust their osmotic potential, such as proline, glycine betaine, soluble sugars, and proteins, which enable the plants to alleviate the osmotic stress and maintain cell turgor, water uptake, and metabolic activity [8,61]. Both salt-sensitive and salt-tolerant poplar species showed an accumulation of free amino acids under long-term SS [62]. Proline is an important osmotic-adjustment substance that exists in a free state in plant cells, has a low molecular weight, is highly soluble in water, is relatively non-toxic, and has no net charge in the physiological pH range [63]. Proline accumulation preserves the osmotic balance under salinity stress, and proline content can be used as a physiological index of plant resistance to SS [8,64]. Salt-tolerant *P. euphratica* increased proline accumulation by 50–90% when exposed to 150–300 mM NaCl [24], while salt-sensitive hybrid poplars, such as *P. alba* cv. *Pyramidalis* × *P. tomentosa*, showed no significant accumulation when exposed to 50 and 150 mM NaCl [65]. Sucrose and total soluble sugars increased with the elevation of foliar Na^+^ and Cl^−^ concentrations in *P. euphratica* [59]. Except for Valine (Val) and Isoleucine (Ile), soluble carbohydrates, sugar alcohols, organic acids, and amino acids in *P. euphratica* leaves did not show significant changes after 24 h of SS. However, the changes of these amino acids were too low to significantly affect the total osmotic potential of leaves [16]. As a “cheap” osmolyte, the accumulation of sodium mainly contributes to osmotic recovery in *P. euphratica* [16,66].

### 3.3. ROS and Reactive Nitrogen Species (RNS)

ROS, including hydrogen peroxide (H_2_O_2_), superoxide anions (O_2_^·^^−^), hydroxyl radicals (^·^OH), and singlet oxygen (^1^O_2_), accumulate when plants are exposed to high SS [67,68]. At moderate levels, functioning as signaling molecules, ROS trigger signal transduction events and elicit specific cellular responses thus regulating plant growth and stress responses [69]. Some ROS can react with almost all the components of living cells leading to severe damage to lipids, proteins, and nucleic acids [70]. Excessive ROS can induce oxidative damage and might be detoxified through enzymatic and non-enzymatic antioxidant systems. Poplars expressing *TaMnSOD* show greatly improved tolerance to NaCl, with higher superoxide dismutase (SOD) activities, lower malondialdehyde (MDA) contents, and lower relative electrical conductivity (REC) than the wild-type lines [71]. The peroxidase (POD) activity of *P. euphratica* was 61.8% higher under 200 mM NaCl stress relative to the control [26], while 3,3′-diaminobenzidine (DAB) staining and H_2_O_2_ measurement demonstrated a sharply increased level of H_2_O_2_ in Chinese white poplar *(Populus tomentosa*) exposed to 200 mM NaCl for 24 h [72]. Most of the genes encoding glutathione peroxidases (GSH-Px), glutathione S-transferases (GST), and glutaredoxins were more highly expressed in salt-stressed *P. tomentosa* than in the control [72], while the transcription levels of genes encoding antioxidant enzymes were upregulated [69] in plants exposed to 150 mM NaCl for 24 h [55]. Genes encoding enzymes involved in the glutathione metabolism pathway, including GSH-Px, glucose-6-phosphate Dehydrogenase (G6PD), glucose phosphate dehydrogenase (GPD), and isocitrate dehydrogenase (IDH), were significantly upregulated in plants treated with NaCl, which facilitated the detoxification of the salinity-induced ROS [15].

Non-enzymatic antioxidants include ascorbate (AsA), glutathione (GSH), and carotenoids (Car). Carotenoids not only protect against active oxygen species by quenching the excited states of photosensitizing molecules and singlet oxygen and by scavenging free radicals, but also protect biomembranes against oxidative damage by modifying the structural and dynamic properties of lipid membranes [73]. Recently, a carotenoid-deficient mutant of bacteria Pantoea sp. YR343 was found, showing reduced colonization on *Populus deltoids* roots [74].

In addition to the antioxidant enzymes, heat-shock transcription factors (HSFs) play a role in scavenging ROS in plants under SS. The transgenic expression of *PeHSF* in tobacco enhanced the activities of ascorbate peroxidase, GSH-Px, and glutathione reductase [75], and *PtHSP17.8* expression in *Arabidopsis* increased the activation levels of antioxidative enzymes under SS [76].

RNS includes nitric oxide (NO^·^), nitric dioxide (NO_2_^·^), nitrous acid (HNO_2_), and dinitrogen tetroxide (N_2_O_4_), which can be produced when plants are subjected to SS. Like ROS, RNS also function as signaling molecules in the response to abiotic stress. NO is involved in plant growth, development, senescence, as well as stress response [69]. NO was also reported to enhance salt tolerance in plants [77]. NO reacts with GSH, forming S-nitrosoglutathione (GSNO); NO, GSNO, and peroxynitrite (ONOO^–^) can produce covalent post-translational modifications (PTMs), such as S-nitrosylation and the protein nitration [78]. However, ONOO^−^, generated from nitric oxide NO and superoxide anion(O_2_^·^^−^), can produce tyrosine nitration of plant proteins and originate nitrosative damage in plant cells [69].

### 3.4. Poplar Salt Stress (SS) Signaling Pathways

Calcium ions (Ca^2+^) are an important secondary messenger in plants and mediate poplar salt tolerance by enhancing Na^+^ exclusion, restricting K^+^ efflux, and sustaining the selectivity of the cell membrane [40]. In higher plants, the Ca^2+^- dependent SOS signaling pathway helps maintain ion homoeostasis and thus confers salt tolerance under saline conditions [79,80]. Upon NaCl exposure, the resulting elevated cytosolic Ca^2+^ levels are sensed by SOS3, which activates SOS2 and stimulates the membrane-localized Na^+^/H^+^ antiporter SOS1, resulting in Na^+^ efflux into the apoplast of the root [79,81]. Similarly, in *Populus*, although *SOS* gene expression is generally ubiquitous, some studies have indicated that SOS2 functions upstream of SOS1 and downstream of SOS3 [82].

In addition to extruding Na^+^ from the roots, SOS1 controls the long-distance transport of Na^+^ and affects its partitioning in plant organs [34,35]. *PeSOS1* (Salt overly sensitive 1 from *P. euphratica*) expression was upregulated 5- to 10-fold in *P. euphratica* leaves treated with 200 mM NaCl for 24 h relative to the untreated controls, and *PeSOS1* partially suppressed salt sensitivity when transgenically expressed in the *Escherichia coli* mutant strain EP432 [83]. Similarly, *PabSOS1* expression was about five times higher after 12 h of NaCl treatment [82]. SOS2 not only acts as a central regulator of Na^+^ extrusion but also is involved in the signaling node between the SOS pathway and other signaling pathways [80].

Tang identified two *CBL10* homologs, *PtCBL10A* and *PtCBL10B*, in the western balsam poplar (*P. trichocarpa*) genome, which may interact with the salt tolerance component PtSOS2 and may help accumulate Na^+^ in vacuoles [84]. Like PtSOS3, PtCBL10s also interacts with PtSOS2 to stimulate the activity of PtSOS1. Whereas PtCBL10s primarily functions in green tissues such as the shoots and targets the downstream component PtSOS2 to the tonoplast, PtSOS3 functions in the roots and targets PtSOS2 to the plasma membrane [84].

**Figure 1:**
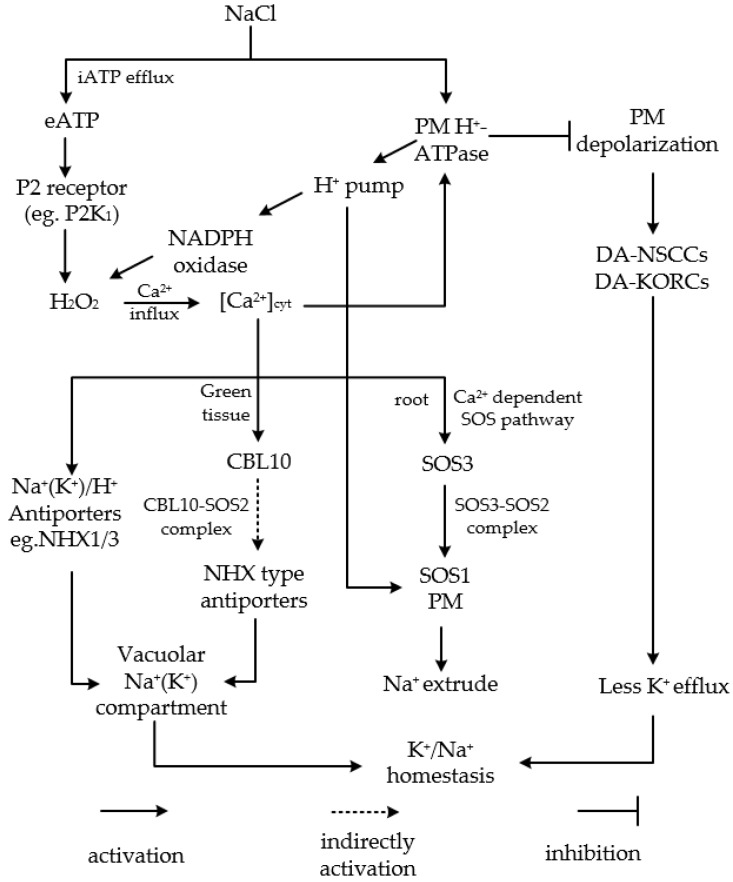
Schematic model showing multiple signaling networks active in *Populus* in response to NaCl stress. NaCl induces the efflux of intracellular ATP (iATP) and an increase in extracellular ATP (eATP), which is sensed by P2K_1_ in the plasma membrane (PM) and leads to the induction of H_2_O_2_ production. This stimulates the movement of Ca^2+^ into the cells via Ca^2+^-permeable channels. The elevated cytosolic Ca^2+^ concentration initiates the SOS pathway by stimulating Na^+^/H^+^ antiporters, such as SOS1, localized in the PM to extrude Na^+^, or activates CBL10, forming the CBL10–SOS2 complex, which may indirectly target the NHX type antiporters to the tonoplast to compartmentalize Na^+^ into vacuoles in green tissues. The elevated cytosolic Ca^2+^ also stimulates tonoplast-localized NHX1/3 to accumulate Na^+^(K^+^) into vacuoles. Besides, the elevated cytosolic Ca^2+^ increases H^+^-ATPase activity in the PM, which activates a H^+^ pump to supply a proton gradient for the Na^+^/H^+^ antiporters, stimulating the extrusion of Na^+^. A proton gradient supplied by the H^+^ pump contributes to the activation of NADPH oxidases, which leads to H_2_O_2_ production. H^+^-ATPases can also inhibit the efflux of K^+^ by further polarizing the PM. All these signaling components help to maintain K^+^/Na^+^ homeostasis in *Populus* cells.

H^+^-ATPases not only provide the proton-motive force used to enhance Na^+^/H^+^ antiporter activity, but also can restrict the NaCl-induced efflux of K^+^ through DA-KORCs and DA-NSCCs (Figure 1). Genes encoding plasma membrane H^+^-ATPases are upregulated in *P. euphratica* [37], likely enhancing the exchange of Na^+^ and H^+^ across the plasma membrane.

As a hinge signal molecule for sensing and responding to SS, H_2_O_2_ is vital for K^+^/Na^+^ homeostasis. In response to SS, the salt-resistant species *P. euphratica* rapidly produces H_2_O_2_ in a process triggered by proton-coupled ion transporters such as the H^+^-pumps and the Na^+^/H^+^ antiporters in the plasma membrane [53]. This H_2_O_2_ accumulation causes a net Ca^2+^ influx by activating non-selective cation channels, which enhances Ca^2+^ concentration in the cytosol [54] and stimulates the SOS signaling pathway [81,85,86] (Figure 1). In addition, H_2_O_2_ signaling results in the upregulation of plasma membrane H^+^-ATPases, whose activity limits the NaCl-induced efflux of K^+^ [40,87]. Overall, H_2_O_2_ is involved in salt resistance in *P. euphratica* by controlling Na^+^ extrusion via the H_2_O_2_–cytosolic [Ca^2+^]–SOS pathway and by reducing K^+^ efflux to maintain ion homeostasis via the H_2_O_2_–Ca^2+^–PM H^+^-ATPases pathway (Figure 1).

NADPH oxidases are the main source of H_2_O_2_. During SS, plasma membrane H^+^-ATPases enhance H^+^ efflux, decreasing the pH and contributing to the activation of NADPH oxidases, which leads to H_2_O_2_ production and triggers the Ca^2+^-dependent SOS signaling pathway [53,54,87] (Figure 1). NaCl induces a transient increase in extracellular ATP (eATP), which is sensed by purinoceptors in the plasma membrane (e.g., P2K_1_) and causes a rapid H_2_O_2_ burst that in turn increases the concentration of Ca^2+^ in the cytosol of *Populus* cells [36,88,89]. Consequently, the salt-elicited eATP-H_2_O_2_-cytosolic [Ca^2+^] cascade contributes to enhancing Na^+^ extrusion through the Ca^2+^-dependent SOS pathways and to reducing K^+^ efflux by activating H^+^-ATPase, thus controlling cellular K^+^/Na^+^ homeostasis (Figure 1).

NaCl-induced expression of *HSF* (Heat shock transcription factor) in *P. euphratica* is markedly restricted by inhibitors of NADPH oxidase and Ca^2+^-permeable channels, suggesting that salt-induced H_2_O_2_ and cytosolic Ca^2+^ enhance the transcription of *HSFs*, which in turn upregulate genes encoding antioxidant enzymes for scavenging ROS under saline conditions [75].

In conclusion, eATP, Ca^2+^, H_2_O_2_, NADPH, H^+^-ATPase, and Na^+^ (K^+^)/H^+^ transporters play important roles in mediating salt tolerance in *Populus* trees.

## 4. Candidate Genes Used for the Genetic Improvement of Salt Tolerance

Currently, many studies are focused on introducing known salt-response signaling genes into *Populus* and testing the performance of the transgenic plants in high-salinity conditions. Many of these genes confer a significantly improved salt tolerance, as described below. Transferring transcription factors (TFs) genes into poplars is generally a more efficient approach than transferring structural genes, because transcription factors usually regulate the expression of many target genes in related pathways. A total of 59 *ERF* (Ethylene response factor) genes are associated with SS in *Populus* [90]. *ERF76* from dihaploid *P. simonii* × *P. nigra* plants was transferred into the same *Populus* clone and significantly upregulated 16 genes encoding other transcription factors, as well as 45 stress-related genes [91]. When exposed to SS, *ERF76*-expressing transgenic plants were significantly taller and had increased root lengths, fresh weights, abscisic acid (ABA) and gibberellic acid (GA) contents compared to the control. Transgenic *ERF76* expression enhanced salt tolerance by upregulating the expression of stress-related genes and increasing ABA and GA biosynthesis [91]. The *PsnERF75* gene from *P. simonii* × *P. nigra* is induced by salt, drought, and ABA treatments [92] and confers salt tolerance when transgenically expressed in *Arabidopsis* [91].

The DREB (for dehydration-responsive element-binding protein) transcription factors, members of the ERF family, are vital regulatory nodes in the signaling pathways involved in the salt-stress response [93]. *PeDREB2a*, encoding a DREB transcription factor in *P. euphratica*, improved salt tolerance in *Arabidopsis* or birdsfoot trefoil (*Lotus corniculatus*) when transgenically expressed under the stress-inducible *rd29A* promoter [94]. The transgenic expression of *LbDREB* (a *DREB* gene from the halophyte *Limonium bicolor*) in *Populus ussuriensis* enhanced its resistance to salt, increasing its SOD and POD activities and the expression of the genes encoding these enzymes, reducing its MDA content, and enhancing its proline accumulation in the leaves [93]. The transgenic *P. ussuriensis* plants also had higher root/shoot ratios, higher relative water contents (RWC), and lower relative electrolytic leakage. Consistent with these changes, the genes encoding NAM (no apical meristem), GT-1(trihelix transcription factor), and WRKY70 (WRKY transcription factor 70) displayed inducible temporal expression patterns and are important components in the SS response signaling networks [93]. The LbDREB protein may inhibit the expression of *NAM*, *GT-1*, and *WRKY70* and induce the expression of *SOD* and *POD* in response to high salinity stress, but this requires further verification.

The GTPase RabE is located in the Golgi apparatus and the plasma membrane, where it plays an important role in vesicle transport [95]. The overexpression of constitutively active *PtRabE1b* conferred salt tolerance in poplar [96]. This gene is directly co-expressed with many genes involved in salt tolerance, such as *HSFA4a*, *SOS2*, *MPK19*, and the Ca^2+^ signaling-related genes *CAM7*, *CKL6*, and calcium exchanger. *HSFA4a* expression is regulated by oxidative stress and MPK3/MPK6 and positively influenced salt tolerance in *Arabidopsis* [97]. *CmHSFA4*, a *Chrysanthemum* homologue of this gene, positively regulates salt tolerance by regulating the activities of SOS1, HKT2, and the ROS scavengers [98].

Recently, Yoon et al. identified a novel gene, *PagSAP1*(stress-associated proteins), from the hybrid poplar *P. alba* × *Populus glandulosa*. *PagSAP1* negatively mediates salt-stress responses, and SS can in turn suppress the expression of this gene in poplar roots [99]. *PagSAP1* overexpression resulted in enhanced sensitivity to SS, while *PagSAP1* silencing via RNA interference (RNAi) significantly increased cytosolic Ca^2+^ in the roots. This increased cytosolic Ca^2+^ activated SOS signal transduction, resulting in high *SOS3* transcript levels in the RNAi-derived plants. *HKT1* expression is significantly reduced in all poplar genotypes under salt treatment; however, the lowest level is observed in the *PagSAP1*-overexpressing lines. HKT1 is responsible for Na^+^ influx and xylem-mediated Na^+^ recirculation from the shoot to the root [100]; therefore, the low HKT1 activity levels in the *PagSAP1*-overexpressing lines may explain the higher Na^+^ accumulation in the leaves and the lower Na^+^ levels in the roots compared with the control and RNAi-derived lines. By contrast, the excess Na^+^ in the roots of the *PagSAP1*-RNAi lines was eliminated by increased SOS1 activity, which resulted in lower Na^+^ levels in both the roots and the leaves of these lines. As a result, the salt tolerance of the *PagSAP1*-RNAi lines was improved through the upregulation of *SOS3*, *SOS1*, *HKT1*, *H^+^-ATPase*, *AAA-type ATPase*, and Arabidopsis K+ channel 2 (*AKT2*), all of which are essential for maintaining Na^+^/K^+^ homeostasis [99].

The poplars SOS proteins share high functional conservation with their *Arabidopsis* homologues [82]. SOS2 interacts with or regulates the activity of several tonoplast-localized transporters, such as the Ca^2+^/H^+^ antiporter [101], the vacuolar H^+^- ATPase [102], and the Na^+^/H^+^ exchanger [103,104]. *PtSOS2.1*, *PtSOS2.2*, and *PtSOS2.3* (the *PtSOS2* genes in *P. trichocarpa*) overexpression improves the salt tolerance of poplars and increases the concentrations of proline and photosynthetic pigments, relative water content, and the activity of their antioxidant enzymes, while significantly decreasing the levels of MDA [105].

The mutant SOS2 protein PtSOS2TD, generated by mutating the 169th amino acid in the activation loop of PtSOS2 from threonine (T) to aspartic acid (D), is more active than PtSOS2 and can sufficiently activate PtSOS1 in a PtSOS3-independent manner [80]. *PtSOS2TD* overexpression in poplars significantly increased salt tolerance, causing higher plasma membrane Na^+^/H^+^ exchange activity, greater Na^+^ efflux, decreased Na^+^ accumulation in the leaves, and improved ROS scavenging capacity [80].

The transgenic expression of the *PtCBL10s* (Calcineurin B-like from *P. trichocarpa*) conferred greater salt tolerance to poplars by maintaining shoot ion homeostasis under SS. The doubling of the *CBL10* genes in poplar may represent an evolutionary adaptation to the adverse environment [84].

The genes that have been shown to increase the salt tolerance of transgenic *Populus* are presented in Table 1. 

## 5. Conclusions and Outlook

Soil salinization is increasingly problematic and is now a dominant factor limiting *Populus* growth [7]. Therefore, it is important to improve the salt tolerance of poplar trees. Plant salt tolerance is a typical quantitative trait affected by many physiological and biochemical factors [15]. Different *Populus* species, such as the salt-resistant poplar species *P. euphratica* and the salt-sensitive *Populus* species *P. × canescens*, have different SS responses [114,115]. Furthermore, trees such as the hybrid poplar 107 display different responses when exposed to different salinity levels [15]. Overall, *Populus* adapt to SS by maintaining suitable Na^+^/K^+^ ratios, accumulating osmotic-adjustment substances, activating antioxidative enzymes and antioxidants, and activating stress response signaling networks to reduce the negative effects of high salinity. With the innovation of transcriptomics technologies, a substantial number of stress-responsive and/or stress-regulated genes have been identified—in addition to the signal regulatory networks in which they function—and transferred between *Populus* and *Arabidopsis* or other species with great success. This has greatly elucidated the molecular mechanisms of the poplar stress responses [113].

ROS/RNS and hormones were identified as signaling molecules involved in the response to SS avoiding high salinity damage. The crosstalk between ROS, RNS, ABA, ethylene, and/or other hormones in poplar salt stress will be further studied. Molecular chaperones, especially dehydrins and osmotin, which contribute to protect proteins, are supposed key factors for coping with SS [16]. Nutrient fertilization with N and P was reported to reduce the accumulation of ROS (e.g., O_3_) and enhance membrane stability, thus protecting from oxidative stress by activating a cross-talk between antioxidant and osmotic mechanisms [116]. The role of mycorrhization and polymer amendment in enhancing mineral nutrition and improving salt tolerance is also a topic of future study. All of the above topics need to be more deeply studied in the future to improve salt tolerance in poplar species as well as in other tree species.

Determining the key transcription factors and molecular mechanisms underlying salt tolerance is an important goal for future research and will facilitate the enhancement of salt tolerance in *Populus*.

## Figures and Tables

**Table 1:** Candidate genes for improving salt tolerance in *Populus*.

Candidate Genes and Source	Transgenic Species	Effect of SS in Transgenic Species Compared with WT	Reference
*AtNHX1/3* (Vacuolar Na^+^/H^+^ antiporter from *Arabidopsis thaliana*)	*Populus davidiana* × *Populus bolleana*	1 Normal growth and morphology; 2 Promoted vacuolar Na^+^ (K^+^)/H^+^ exchange activity; 3 Increased Na^+^ and K^+^ accumulation in the vacuoles; 4 Elevated the eaccumulation of proline.	[47]
*AtNHX1* (See above)	*Populus × euramericana* ‘Neva’	1 Enhanced plant growth and photosynthetic capacity; 2 Lowerd MDA and REC; 3 Increased Na^+^ (K^+^) accumulation in roots and leaves.	[44]
	*Populus × euramericana* ‘Neva’	1 Reduced decrease in Chl, Car, PSII, Fv/Fm, and qP; 2 Smaller reduction of Pn, Gs, Ci, CE; 3 Greater increase of stem and leaf, smaller increase in root.	[43]
	*Populus deltoides* × *P. euramericana* CL ‘NL895′	1 Higher content of sodium ions; 2 Decreased MDA content.	[45]
*PtSOS2TD* (Salt overly sensitive *2* from *Populus trichocarpa*)	*P. davidiana × P. bolleana* hybrid poplar clone Shanxin	1 More vigorous growth; 2 Greater biomass produced; 3 Less Na^+^ in the leaves; 4 Higher Na^+^/H^+^ exchange activity and Na^+^ efflux; 5 More scavenging of ROS.	[80]
*PtSOS2* (See above)	*Populus tremula × Populus tremuloides* Michx clone T89	1 Improved PM Na^+^/H^+^ exchange activity, Na^+^ efflux; 2 Higher proline activity; 3 Higher RWC and sustained decrease of water loss; 4 Increased SOD, POD, CAT activity; 5 Decreased MDA concentration.	[105]
*PtCBL10A* and *PtCBL10B* (Calcineurin B-like from *P. trichocarpa*)	*P. davidiana × P. bolleana* hybrid poplar clone Shanxin	1 Less impairment by SS with higher stature and greater shoot biomass; 2 Lower Na^+^ in the leaves, more Na^+^ in the stem.	[84]
*PeCBL6*, *PeCBL10* (Calcineurin B-like from *P. euphratica*)	triploid white poplar	1 Higher height growth rate; 2 Less wilted leaves; 3 Lower MDA content; 4 Higher chl content.	[106]
*PtSOS3* (Salt overly sensitive *3* from *P. trichocarpa*)	*P. davidiana × P. bolleana* hybrid poplar clone Shanxin	1 Lower Na^+^ in the root; 2 Higher K^+^ content in the root; 3 More Na^+^ in the stem.	[84]
*TaMnSOD* (Mn-superoxide dismutases from *Tamarix Androssowii*)	*P. davidiana × P. bolleana* hybrid poplar clone Shanxin	1 Higher SOD activity; 2 Lower MDA contents; 3 Lower REC; 4 More weight gains.	[71]
*TaLEA* (Late embryogenesis abundant from *T. androssowii*)	*Populus simonii* × *Populus nigra* Xiaohei poplar	1 Decrease in MDA content; 2 Decrease in relative electrolyte leakage; 3 Improved salt and drought resistance.	[107]
	*P. davidiana × P. bolleana*	1 Higher Survival percentages; 2 Higher Seedling height and photosynthetic capabilities; 3 Lower Na^+^ in young leaves but higher in yellow and withered leaves.	[108]
*ERF76* (Ethylene response factor from di-haploid *P. simonii × P. nigra*)	*P. simonii × P. nigra* di-haploid	1 Higher plant height, root length, fresh weight; 2 Higher in ABA and GA concentration.	[91]
*JERFs* (Jasmonic ethylene responsive factor from the tomato)	*Populus alba × Populus berolinensis*	1 Lower reductions of height, basal diameter, and biomass; 2 Lower reduction in leaf water content and increase in root/crown ratio; 3 Greater increase of foliar proline concentration; 4 Higher foliar Na^+^ concentration.	[109]
*LbDREB* (dehydration responsive element binding TF from *Limonium bicolor*)	*Populus ussuriensis* Kom. Chinese Daqing poplar	1 Higher SOD, POD activity; 2 Less MDA accumulation in the leaves; 3 More proline accumulation; 4 Increased root/shoot ratio; 5 Reduced decrease of RWC; 6 Lower increase of relative electrolytic leakage.	[93]
*AhDREB1* (dehydration responsive element binding-like TF from the halophyte *Atriplex hortensis*)	*Populus tomentosa*	1 Higher survival rate; 2 High proline content.	[110]
*AtSTO1* (Salt tolerant1 from *Arabidopsis thaliana*)	*P. tremula × P. alba* Poplar 717-1B4	1 Higher aboveground biomass; 2 Higher root biomass; 3 Higher shoot height; 4 Higher chl content.	[111]
*AtPLDα* (Phospholipase Dα from *A. thaliana*)	*P. tomentosa*	1 Higher root rate and root length; 2 Reduced decrease of total chl content; 3 Lower REC and MDA content 4 Higher SOD, POD, and CAT activities.	[112]
*AtSRK2C*, *AtGolS2* (Stress responses, SNF1-related protein kinase 2C, galactinol synthase *2* from *A. thaliana*)	*P. tremula × tremuloides*	1 Reduced decrease of dry weight; 2 Reduced decrease of total adventitious root length.	[113]
*PtRabE1b*(Q74L) (Rab GTPase from *P. trichocarpa*)	*P. alba × P. glandulosa* clone 84 K	1 More adventitious roots; 2 Greater root growth status in seedlings.	[96]
*PagSAP1* (stress-associated proteins from *P. alba × P. glandulosa*)	*P. alba × P. glandulosa*	RNAi plants accumulate more Ca^2+^, and K^+^ and less Na^+^.	[99]

SS, salt stress; PM, plasma membrane; SOD, superoxide dismutase; POD, peroxidase; CAT, catalase; MDA, malondialdehyde; Chl, chlorophyll; Car, carotenoid; PSII, actual quantum yield of PSII; Fv/Fm, maximum photochemical efficiency; qP, photochemical quenching coefficient; Pn, net photosynthetic rate; Gs, stomatal conductance; Ci, internal CO_2_; CE, carboxylation efficiency concentration; ROS, reactive oxygen species; REC, relative electrical conductivity; RWC, relative water content; ABA, abscisic acid; GA, gibberellic acid.

## Abbreviations

SOS	salt overly sensitive
ROS	reactive oxygen species
SOD	superoxide dismutase
POD	peroxidase
MDA	malondialdehyde
GSH-Px	glutathione peroxidases
GST	glutathione S-transferases
GR	glutathione reductase
GPX	glutathione peroxidases
GPD	glucose phosphate dehydrogenase
G6PD	glucose-6-phosphate dehydrogenase
APX	ascorbate peroxidase
IDH	Isocitrate dehydrogenase
GA	gibberellic acid
ABA	abscisic acid
REC	relative electrical conductivity
RWC	relative water content
TFs	transcription factors
Chl	chlorophyll
Pn	net photosynthetic rate
Gs	stomatal conductance
Ci	internal CO_2_
CE	carboxylation efficiency concentration;
Car	carotenoid
PSII	actual quantum yield of PSII
qP	photochemical quenching coefficient
NPQ	non photochemical quenching
Fv/Fm	maximum photochemical efficiency
eATP	extracellular ATP
iATP	intracellular ATP
DAB	3,3′-diaminobenzidine
PM	plasma membrane
D A	depolarization activated
KORCs	K^+^ outward rectifying channels
NSCCs	non-selective cation channels
SS	salt stress
